# *Enterococcus faecium* Alleviates Gut Barrier Injury in C57BL/6 Mice with Dextran Sulfate Sodium-Induced Ulcerative Colitis

**DOI:** 10.1155/2021/2683465

**Published:** 2021-11-25

**Authors:** Wei He, Weijie Ni, Junning Zhao

**Affiliations:** ^1^Department of Gastroenterology, Geriatric Hospital of Nanjing Medical University, Nanjing 210024, China; ^2^School of Medicine, Southeast University, Nanjing 210009, China

## Abstract

The involvement of gut microbiota composition in ulcerative colitis is strongly supported by previous research. Growing evidence suggests that probiotic therapy protects against inflammatory bowel disease in animal models and patients. However, as a probiotic, the role of *Enterococcus faecium (E. faecium)* in UC remains unclear. Nevertheless, the potential mechanism of the protective effect of *E. faecium* remains unknown. In this study, a dextran sulphate sodium-induced (DSS-induced) colitis model was used to detect the underlying mechanism of *E. faecium* in maintaining gut homeostasis. ELISA was performed to detect the levels of cytokines (TNF-*α*, IL-1*β*, IL-6, and IL-10). Furthermore, 454 pyrosequencing was used to investigate the microbiota composition in fecal samples. The results illustrate that *E. faecium* administration could prevent DSS-induced gut inflammation and intestinal flora imbalance. At the same time, the damage to intestinal mucosal barrier and tight junctions was partially repaired. These results demonstrate the preventive effect of *E. faecium* in DSS-induced intestinal injury. The present study provides new insights into the medicinal value of *E. faecium* for UC.

## 1. Introduction

Inflammatory bowel disease (IBD) is complex and presents many challenges to gastroenterologists [1]. Ulcerative colitis (UC) is a major form of IBD, which is marked by aberrant inflammation within the human colon [2]. Previous studies suggest that microbiota dysbiosis and intestinal barrier dysfunction are closely related to UC [3].

Although there have been many studies on UC, its pathological mechanism remains poorly understood [4]. Previous research has demonstrated that epithelial barrier and mucous barrier defects are strongly related to the pathogenesis of UC [5]. Furthermore, Suzuki indicated that the expression of occludin and zonula occludens-1 (ZO-1), which are tight junction proteins, is associated with gut permeability [6]. Ma et al. confirmed that the expression level of ZO-1 is negatively correlated with the degree of UC [7]. Recent studies have shown that microbiota could have an impact on intestinal permeability [8].

The intestinal microbiota, which seems to be a hidden organ composed of trillions of microorganisms, makes essential contributions to intestinal homeostasis [9]. Probiotics are defined as nonpathogenic living microorganisms that are beneficial to disease treatment or host health. A series of studies confirmed that imbalance of intestinal microflora refers to the pathogenesis of UC [3]. *Enterococcus faecium* (*E. faecium*) is a type of probiotic that inhabits the human gastrointestinal tract [10]. A previous study showed that regular intake of Enterococcus faecium CRL 183 could reduce the DSS-induced colitis injury on rats [11, 12]. Consistent with the effect in *Caenorhabditis elegans*, Pedicord et al. observed that *E. faecium* colonization improves host intestinal epithelial defense programs in mice [13]. Increasing evidence has demonstrated that *E. faecium* is associated with gut protection. In this study, the relationship between *E. faecium* administration and gut barrier integrity was analyzed.

The protective mechanism of probiotics against UC is multifaceted. The inhibition of intestinal pathogenic bacteria by probiotics plays a crucial role in the protective effect of probiotics in UC. Although previous studies have illustrated that *E. faecium* could have gut-protective effects [11, 12], there are still studies showing controversial results [13]. It is of great value to explore the mechanism of *E. faecium* in UC.

In this work, we investigated the role of E. faecium on dextran sulphate sodium-induced (DSS-induced) intestinal injury in order to provide new methods for UC treatment.

## 2. Materials and Methods

### 2.1. Animals

Male C57BL/6 mice (body weight, 22 to 24 g; 7–8 weeks old) were purchased from Shanghai Jiesijie Experimental Animal Co., Ltd. (Pujiang, Shanghai, China). Before the experiment, animals were adaptively fed for a week. During the experiment, the mice were weighed daily to observe the changes in body weight. All mice were maintained under standard conditions in an animal house at Nanjing Medical University. Experimental ulcerative colitis was induced by dextrose sulfate sodium (DSS, 3% wt/vol, dissolved in ddH2O, administered from day 1 to day 7). Mice were randomly chosen to receive different treatments via gavage from day 1 to day 7, including *E. faecium* (R026, Beijing Hanmi Pharm Co., Ltd., Beijing, China, 1 × 10^9^ CFU/day, *n* = 16) and phosphate-buffered saline (PBS) (*n* = 16). On the eighth day, the bleeding scores and colon length were assessed. Half of the mice were sacrificed on day 8, and the other mice were sacrificed on day 14 to observe the effect of *E. faecium* on survival. All animal studies were approved by the Institutional Animal Care and Use Committee of Nanjing Medical University.

### 2.2. Stool Consistency and Bleeding Score

Stool consistency was assigned grades according to the following criteria: normal stool consistency with negative hemoccult: 0, soft stools with positive hemoccult: 1, very soft stools with traces of blood: 2, and watery stools with visible rectal bleeding: 3.

Bleeding score was assigned grades according to the following criteria: normal stool consistency with negative hemoccult: 0, soft stools without positive hemoccult: 1, soft stools with positive hemoccult: 2, very soft stools with traces of blood: 3, and watery stools with visible rectal bleeding: 4.

### 2.3. Colon Pathology and Immunohistochemistry

Colons from mice were excised, flushed, and collected. Colon tissue was fixed in Bouin's fluid (Sigma-Aldrich) and embedded in paraffin. Five micrometer sections of paraffin-embedded colons were prepared for further experiments. Alcian blue staining and HE staining were used to examine mucosal damage. For immunofluorescence (IF), the sections were incubated with anti-ZO-1 antibody (1 : 200 Abcam; Cambridge, MA) and anti-claudin antibody (1 : 200 Abcam; Cambridge, MA) at 4°C. All the results were observed by microscopy. The above histological analysis was performed in a blinded manner to avoid bias.

### 2.4. Intestinal Permeability

Disaccharide permeability probes (50 mg/ml mannitol and 100 mg/ml lactulose) were used to detect intestinal permeability. DSS-treated mice administered phosphate-buffered saline or *E. faecium* (*n* = 4; 4 days) were given a probe after fasting for 12 h by oral gavage, and urine was collected after 12 h. High-performance liquid chromatography was performed to measure the urine concentrations of lactulose and mannitol. The recovery rates of lactulose and mannitol were examined to evaluate intestinal permeability.

### 2.5. Short-Chain Fatty Acid (SCFA) Assay

Fecal samples were collected from the metabolism cage on the 8th day after DSS treatment. After mixing with distilled water, the fecal samples were centrifuged (2,500 x g). The samples were then extracted with ether and sulfuric acid. The ether phase was collected and used to examine the total SCFA concentrations by an Agilent 6890 N gas chromatography machine (Agilent Technologies CA USA).

### 2.6. Serum LPS and Cytokines

Blood samples were collected from the left ventricle on the 8th day after DSS treatment. A protein-based enzyme-linked immunosorbent assay (Elisa) was used to detect the levels of cytokines (RAB0274; RAB0477; RAB0308; RAB0245, Merck, USA). In brief, after the antibody was coincubated overnight with the plate, the sample to be tested was added, followed by a reaction solution for coloration, and the level of cytokine was detected by colorimetry. Limulus test kit (EC644405; Xiamen Houshiji, Ltd., Xiamen, China) was used to detect LPS.

### 2.7. Microbiological Analysis of Fecal Samples

454 pyrosequencing was used to determine the microbiota composition by targeting the V3-V4 region of the bacterial 16S rRNA gene. Sequences were binned for a minimal sequence length of 300 bp, a minimal base quality threshold of 30 cycles, and a maximum homopolymer length of 6 cycles. Sequences were further clustered into operational taxonomic units (OTUs) or phylotypes. According to the results, Seqmatch and Blastall were used to assign the closest taxonomic neighbors and relative bacterial species.

### 2.8. Statistical Analysis

Data are presented as the mean ± SD. Between-group comparisons were performed by one-way ANOVA followed by Dunnett's multiple comparison test or Student's *t*-test. Differences with *P* < 0.05 were considered statistically significant.

## 3. Results

### 3.1. *E. Faecium* Treatment Reduced Lethality and Alleviated Intestinal Injuries

**Figure 1:**
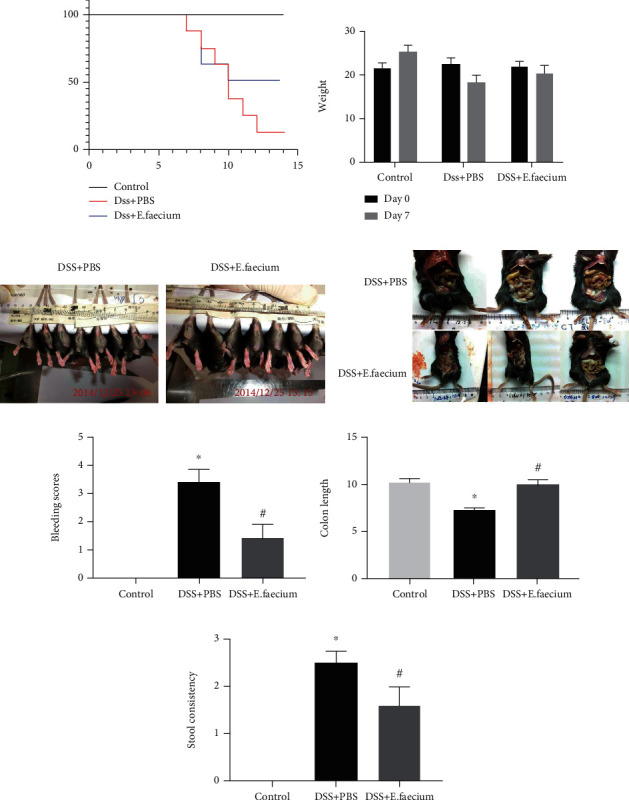
The protective effect of E. faecium against DSS-induced colitis. (a) The survival curve for DSS-induced colitis. (b) Weight loss. (c) Typical image of gut damage. (d) Typical image of colon length. (e) Bleeding scores. (f) Colon length. (g) Stool consistency. ^∗^*P* < 0.05 vs. control group; #*P* < 0.05 vs. DSS + PBS group.

DSS-mediated colitis was used to detect the mechanisms of *E. faecium* in protecting the intestine from injury. As shown in Figure 1(a), treatment with *E. faecium* reduced the lethality rate remarkably compared with the DSS + PBS group. Consistent with the survival curve, mice treated with *E. faecium* lost significantly less body weight after DSS treatment (Figure 1(b)). To further detect intestinal injuries in mice, we examined the intestinal tracts and anuses among the three groups. As shown in Figures 1(c) and 1(e), compared with the control group, the bleeding and anabrosis in the anus and intestinal tract of mice were alleviated by *E. faecium* treatment. Meanwhile, DSS induced colon necrosis and shorter colons, which were restored by *E. faecium* treatment (Figures 1(d) and 1(f)). Evaluation of stool consistency also demonstrated the therapeutic effect of *E. faecium* (Figure 1(g)). These results indicate that *E. faecium* administration prevents DDS-induced colon damage in vivo.

### 3.2. *E. Faecium* Treatment Alleviated DSS-Induced Intestinal Mucosal Damage

**Figure 2:**
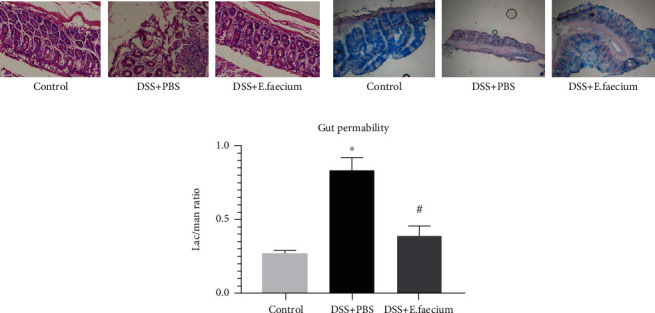
E. faecium restores intestinal mucosal damage induced by DSS. (a) HE staining of the colon. (b) Alcian blue staining of the colon. (c) Detection of intestinal permeability. ^∗^*P* < 0.05 vs. control group; #*P* < 0.05 vs. DSS + PBS group.

As the above research shows, *E. faecium* may protect against DSS-mediated colitis. Previous studies have shown that intestinal mucosal damage is an important phenotype in DSS-induced intestinal injury. To investigate the intestinal mucosal after *E. faecium* treatment, we performed HE staining and alcian blue staining. As shown in Figures 2(a) and 2(b), compared with the control group, inflammatory cell infiltration increased significantly after DSS treatment, and this change was reversed by *E. faecium*. In addition, the levels of mucins were upregulated in the *E. faecium*-treated group. Furthermore, intestinal permeability was measured to test the effect of *E. faecium* administration on DSS-induced intestinal mucosa damage. As shown in Figure 2(c), compared with the DSS + PBS group, the injury of intestinal permeability was alleviated by *E. faecium* treatment. These results suggest that treatment with *E. faecium* may prevent intestinal mucosa damage.

### 3.3. *E. Faecium* Treatment Helps to Restore Tight Junctions

**Figure 3:**
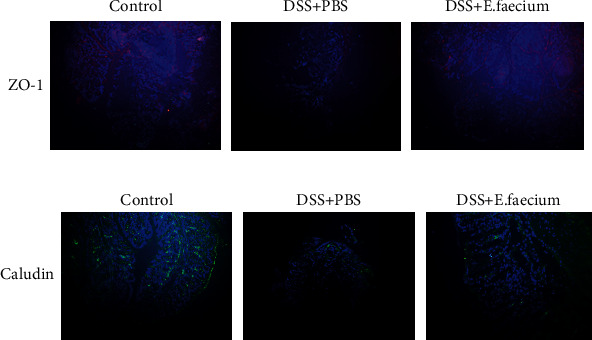
E. faecium protects against intestinal injury by strengthening tight junctions. Immunohistochemical staining of (a) ZO-1 and (b) claudin in the colon.

To further explore the effect of *E. faecium* on the repair of tight junctions, we performed immunofluorescence to detect the expression and distribution of ZO-1 and claudins, which are tight junction-associated markers. As shown in Figure 3, ZO-1 and claudins increased after *E. faecium* treatment. This result indicated that *E. faecium* was involved in the repair of the tight junction complex.

### 3.4. *E. Faecium* Treatment Protected against DSS-Induced Systemic Inflammation

**Figure 4:**
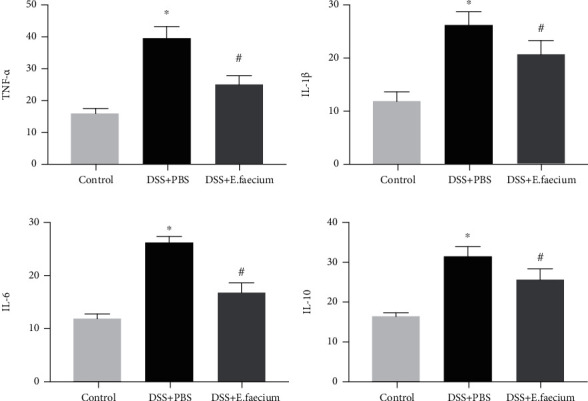
E. faecium treatment alleviates gut inflammation. Inflammatory cytokines: (a) TNF-*α*, (b) IL-1*β*, (c) IL-6, and (d) IL-10. ^∗^*P* < 0.05 vs. control group; #*P* < 0.05 vs. DSS + PBS group.

A previous study demonstrated that the relief of inflammation is related to intestinal permeability. Therefore, we tested serum samples from mice by ELISA to assess the protective effect of *E. faecium* treatment on gut inflammation. Compared with the control group, the levels of plasma cytokines, including TNF-*α*, IL-1*β*, IL-6, and IL-10, were significantly decreased in the *E. faecium*-treated group (Figure 4).

### 3.5. *E. Faecium* Treatment Regulated Anti- and Proinflammatory Factors

**Figure 5:**
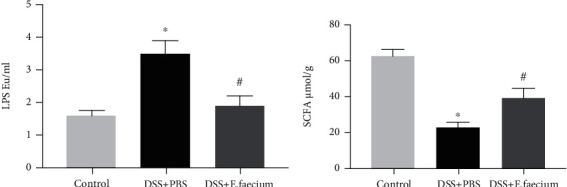
E. faecium treatment shifts the balance of inflammatory factors. The levels of (a) LPS and (b) SCFAs. ^∗^*P* < 0.05 vs. control group; #*P* < 0.05 vs. DSS + PBS group.

To further identify the potential mechanism of *E. faecium* in DSS-induced intestinal inflammation, the plasma LPS of mice was analyzed. As shown in Figure 5(a), the induction of LPS by DSS was reversed by *E. faecium* treatment. Furthermore, compared with the control group, the concentration of total SCFAs was significantly upregulated after *E. faecium* treatment (Figure 5(b)).

### 3.6. *E. Faecium* Treatment Restored the Imbalance of Intestinal Flora Induced by DSS

**Figure 6:**
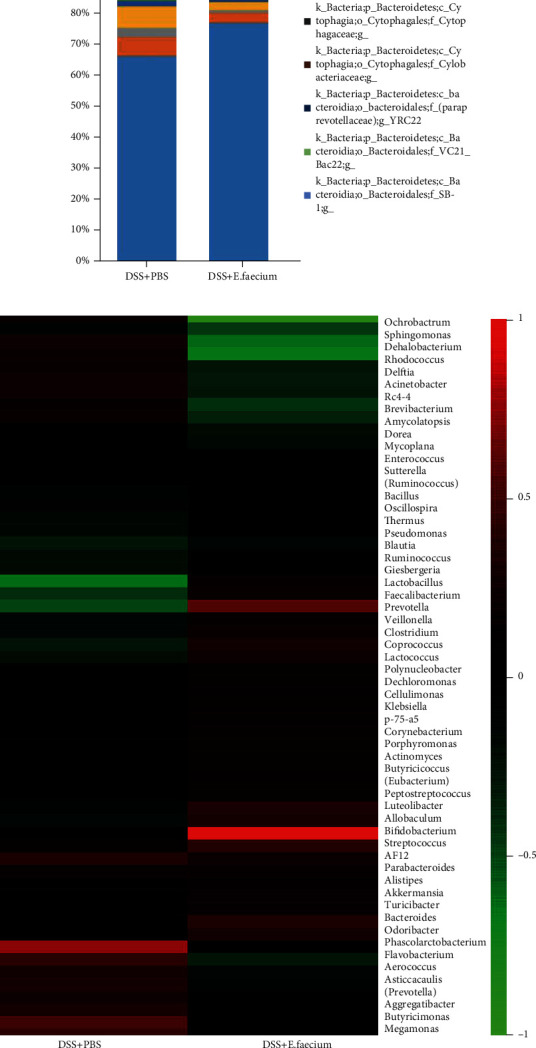
E. faecium treatment restores the imbalance of intestinal flora. (a) Microbiota composition in mice. (b) Heatmap of the abundance of bacterial genera in mice.

Trillions of microorganisms colonize the human gut. These microorganisms are related to regulating the immune system and balancing the internal environment. Therefore, in this study, we performed 16S rDNA sequencing analysis to detect changes in the microbiota after *E. faecium* treatment. As shown in Figure 6(b), compared with the control group, the gut microbiota was significantly altered after *E. faecium* treatment. Specifically, the proportion of *Ochrobactrum* sp. and *Acinetobacter* sp. was reduced upon *E. faecium* treatment. Meanwhile, the proportion of *Butyricicoccus* sp. *Lactobacillus* sp., and *Bifidobacterium* sp. was upregulated. These results revealed the pivotal role of *E. faecium* in restoring the imbalance of intestinal flora induced by DSS.

## 4. Discussion

In this study, the effect of *E. faecium* treatment on a DSS-induced UC model was investigated. The remarkable finding of this study was that *E. faecium* markedly protected against intestinal damage induced by DSS and significantly decreased mortality. Based on alcian blue staining and gut permeability, *E. faecium* treatment contributed to intestinal mucosal injury. Meanwhile, the upregulation of ZO-1 and claudin confirmed that *E. faecium* was able to restore intestinal barrier function by increasing the concentration of tight junction proteins. Consistent with previous studies, *E. faecium* treatment reduced the intestinal damage induced by DSS, which further supports the hypothesis that *E. faecium* colonization improves host intestinal epithelial defense programs in rats [11]. Preview studies have demonstrated that the levels of TNF-*α*, IL-1*β*, IL-6, and IL-10 are upregulated in patients with IBD [14–16]. In the present study, our results also showed that the expression of TNF-*α*, IL-1*β*, IL-6, and IL-10 was downregulated after *E. faecium* treatment.

Increasing evidence has confirmed the anti-inflammatory functions of SCFAs. Previous studies have suggested that SCFA-producing bacteria are significantly downregulated in pediatric CD patients [16]. A study of metabolism and intestinal flora demonstrated that the reduction in SCFA-producing bacteria can result in metabolic stress in inflammatory disease [17]. *E. faecium* treatment increased the levels of SCFAs. Moreover, our data also showed that *E. faecium* treatment can restore the inhibition of *Butyricicoccus* sp., which contributes to butyrate generation and was decreased in IBD [18, 19].

Preview studies suggest that *Ochrobactrum* sp. and *Acinetobacter* sp. were increased in UC patients, while *Lactobacillus* sp., *Butyricicoccus* sp., and *Bifidobacterium* sp. were decreased [20–24]. The evidence presented so far implies that a balance of healthy microbiota is required to shape the immune system response during disease [25]. Meijer et al. indicated that beneficial species of bacteria, including *Lactobacillus* sp. and *Bifidobacterium* sp., can prevent or attenuate UC [26]. In this study, our results indicated that, compared with the control group, beneficial bacteria, including *Lactobacillus* sp., *Butyricicoccus* sp., and *Bifidobacterium* sp., were upregulated in the *E. faecium*-treated groups, demonstrating the potential therapeutic value of *E. faecium*.

**Figure 7:**
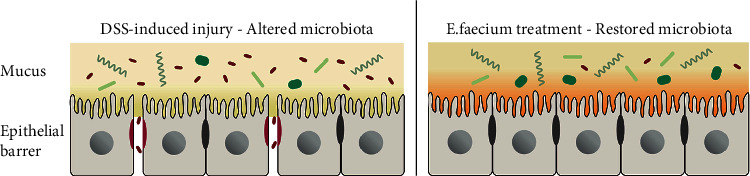
Working model of E. faecium treatment. E. faecium treatment improved the intestinal flora, thereby protecting the intestinal mucosa and reducing the damage to tight junctions.

In conclusion, in this study, it was confirmed that *E. faecium* administration prevents the intestinal mucosa and reduces damage to tight junctions, as summarized in Figure 7. *E. faecium* could reduce intestinal damage and improve the imbalance of the intestinal flora induced by DSS. This study explored the potential mechanisms of *E. faecium* treatment and provided new insight into the intervention and treatment of UC.

## Data Availability

We declare that the data used to support the findings of this study are available from the corresponding author upon request.
